# *Mencius* gen. nov. (Hemiptera, Cicadellidae, Ledrinae, Ledrini), a new leafhopper genus from South China

**DOI:** 10.3897/zookeys.1286.175302

**Published:** 2026-07-29

**Authors:** Yu-Jian Li, Li-Na Jiang, Ren-Huai Dai

**Affiliations:** 1 School of Life Sciences, Qufu Normal University, Qufu, Shandong Province 273165, China Institute of Entomology, Guizhou University Guiyang China; 2 Shandong Key Laboratory of Wetland Ecology and Biodiversity Conservation in the Lower Yellow River, Qufu, Shandong Province 273165, China School of Life Sciences, Qufu Normal University Qufu China; 3 Institute of Entomology, Guizhou University, Guiyang, Guizhou Province 550025, China Shandong Key Laboratory of Wetland Ecology and Biodiversity Conservation in the Lower Yellow River Qufu China

**Keywords:** Auchenorrhyncha, ledrine leafhoppers, morphology, new combination, new genus, new species, taxonomy

## Abstract

A new leafhopper genus, *Mencius***gen. nov**., is established to accommodate *Mencius quinquespinosus***comb. nov**. and *Mencius simprocessus***sp. nov**., the latter described from southern China. The new genus is compared with the similar genera *Petalocephala* Stål and *Platycephala* Kuoh. A key to distinguish *Mencius* from these genera is provided. Morphological descriptions, illustrations, and distribution data for the new taxa are presented.

## Introduction

The leafhopper subfamily Ledrinae constitutes a distinctive group within Cicadellidae, characterized by numerous prominent and unique morphological features (Jones and Deitz 2009). With a predominantly tropical and subtropical distribution, this subfamily currently comprises 71 genera and 462 valid species worldwide (according to the World Auchenorrhyncha Database (Dmitriev et al. 2022 onward). China is one of the major diversity centers for Ledrinae, with more than 180 species classified in 30 genera (Li et al. 2023; Wang et al. 2024; Li et al. 2025; Li et al. 2026).

During ongoing taxonomic studies of Chinese ledrine leafhoppers, we discovered specimens representing a previously unrecognized genus exhibiting morphological similarities to *Petalocephala* Stål and *Platycephala* Kuoh. This new genus is described here as *Mencius* gen. nov., including one new species, *M. simplex* sp. nov., and one new combination, *M. quinquespinosus* comb. nov. Comprehensive descriptions, illustrations, and diagnostic comparisons are provided.

## Material and methods

Specimens examined in this study were collected from Guangxi, China. Morphological terminology follows Dietrich (2005) and Jones and Deitz (2009). Specimens were studied using a Leica MZ 12.5 stereomicroscope. Body length was measured from the apex of the head to the apex of the forewings at rest. Male genitalia were dissected, cleared in 10% KOH, and examined in glycerin. Illustrations were prepared manually using a drawing tube attached to the stereomicroscope. Photographs were taken with a Leica D-Lux 3 digital camera. Images were processed and arranged using Adobe Photoshop CS6. All specimens are deposited in the Insect Collection of the School of Life Sciences, Qufu Normal University, Qufu, China (**QFNU**).

## Results

### Key to known genera of Ledrinae from China

*Lataramus* Wang, Li, Dietrich & Dai is not included in the key, because according to the description in Wang et al. (2024), this genus should be a synonym of *Platycephala* Kuo; we will conduct further research to confirm our viewpoint.

**Table:** 

1	The length of the crown is approximately equal to the width, and the side edge of the crown protrudes like a circular arc	***Digitata* Wang, Li & Dai**
–	Not as above	**2**
2	Pronotum lateral margins straight and parallel to each other	***Parallelalatus* Wang, Dietrich & Dai**
–	Pronotum lateral margins not parallel to each other	**3**
3	Tibia of hindfoot flat, foliaceous	**4**
–	Tibia of hindfoot not foliaceous	**11**
4	Pronotum usually prominent, humped-like; with lateral extensions	**5**
–	Pronotum often declivous or weakly prominent; without extensions but lateral area concave	**6**
5	Lateral edge of pronotum laminately and subangularly dilated	***Eleazara* Distant**
–	Lateral edge of pronotum straight	***Complanledra* Cai & He**
6	Lateral area of pronotum with ear-shaped protrusions or longitudinal ridges	***Ledra* Fabricius**
–	Not as above	**7**
7	Crown significantly prolonged, middle length of crown greater than width between eyes	***Ledropsis* White**
–	Medium length of crown less than width between eyes	**8**
8	Chinese characters “山” shaped ridge on crown prominent	**9**
–	Crown no ridge or weak	***Confucius* Distant**
9	Forewing without a large, more or less developed, sclerotized tubercle at first split of M vein	***Paraconfucius* Cai**
–	Forewing with a large, more or less developed, sclerotized tubercle at first split of M vein	**10**
10	Crown with 2 window-like patches; bend at end of style with a small protuberance	***Funkikonia* Kato**
–	Crown without window-like patch; bend at end of style without a small protuberance	***Kuohledra* Cai & He**
11	Forewing cells strongly depressed, forewing veins raised	***Dusuna* Distant**
–	Not as above	**12**
12	Lateral edge of pronotum protrudes in an angular shape	**13**
–	Lateral edge of pronotum does not protrude in an angular shape	**17**
13	Pronotal lateral extensions broad, with margins subtriangular	**14**
–	Pronotal lateral extensions broad, with margins rounded	***Thlasia* Germar**
14	Body large, length longer than 19 mm; style with long fine setae on inner edge	***Macrotrichia* Zhang, Sun & Dai**
–	Style without long fine setae on inner edge	**15**
15	Pronotum with two regions near the anterior margin foveate and the posterior region gibbous; hind femur distal chaetotaxy 2 + 0; male subgenital plate considerably longer than pygofer	***Paratituria* Li & Cao**
–	Pronotum almost flat, with the angular projections directed laterad; hind femur distal chaetotaxy 2 + 1; subgenital plate extends only slightly beyond pygofer apex	**16**
16	Lateral extensions of pronotum broad and well developed	***Tituria* Stål**
–	Lateral extensions of pronotum narrow and not well developed	***Neotituria* Kato**
17	Body small, average length 6–9 mm	**18**
–	Body mid-large, length longer than 9 mm	**21**
18	Center of crown with a longitudinal groove	***Petalocephaloides* Kato**
–	Center of crown with a longitudinally ridged or flat	**19**
19	Center of crown flat; base of forewing A veins not raised	**20**
–	Center of crown with a longitudinally ridged; base of forewing A veins raised	***Parapetalocephala* Kato**
20	Aedeagus longitudinally flat or slender, with ventral process	**27**
–	Aedeagus slender, without ventral process	***Arenoledra* Kuoh**
21	Body stout; style with an odontoid process on the outside of the bend near end	**22**
–	Body slender; style without odontoid process on the outside near end	**23**
22	Forewing terminal venation reticulate; aedeagus slender	***Destinoides* Cai & He**
–	Forewing terminal venation not reticulate; aedeagus longitudinally flattened	***Destinia* Nast**
23	Crown significantly wider than the front of pronotum; pygofer posterior margin concave	***Laticorona* Cai**
–	Crown not significantly wider than pronotum; pygofer posterior margin not concave	**24**
24	Crown broadly rounded; aedeagus slender, terminal with 2 pairs of processes	***Pachyledra* Schumacher**
–	Crown parabolic; aedeagus without process or with 1 process or with 1 pair of processes	**25**
25	Aedeagal gonopore apical, orifice with dorsal slender processes	***Mencius* gen. nov**.
–	Aedeagal orifice without dorsal slender process	**26**
26	Lateral process on aedeagus paired	***Platycephala* Kuoh**
–	Aedeagus without lateral process	***Petalocephala* Stål**
27	Male pygofer without long ventrocaudal process, gradually narrowing from base to apex, apex narrow and curved dorsally, and with a small process on apical half of ventral margin	***Dietrichiana* Jiang, Dai & Li**
–	Not as above	**28**
28	Aedeagus longitudinally flat	***Midoria* Kato**
–	Aedeagus slender	**29**
29	Pygofer with some small stout setae subapically	***Parathlasia* Li, Jiang, Li & Xing**
–	Pygofer without some small stout setae subapically	***Multiprocessa* Wang, Li, Dietrich & Dai**

#### Mencius

Taxon classification

Animalia

HemipteraCicadellidae

Li, Jiang & Dai gen. nov.

1CB4572B-27AF-5FAD-A888-065C2BE78644

https://zoobank.org/7215F6BF-79D2-4532-AAA4-0A6C1951D2EC

Figs 1, 2, 3, 4, 5

##### Type species.

*Petalocephala quinquespinosa* Wang, Yang, Li & Li, 2024

**Figure 1.**
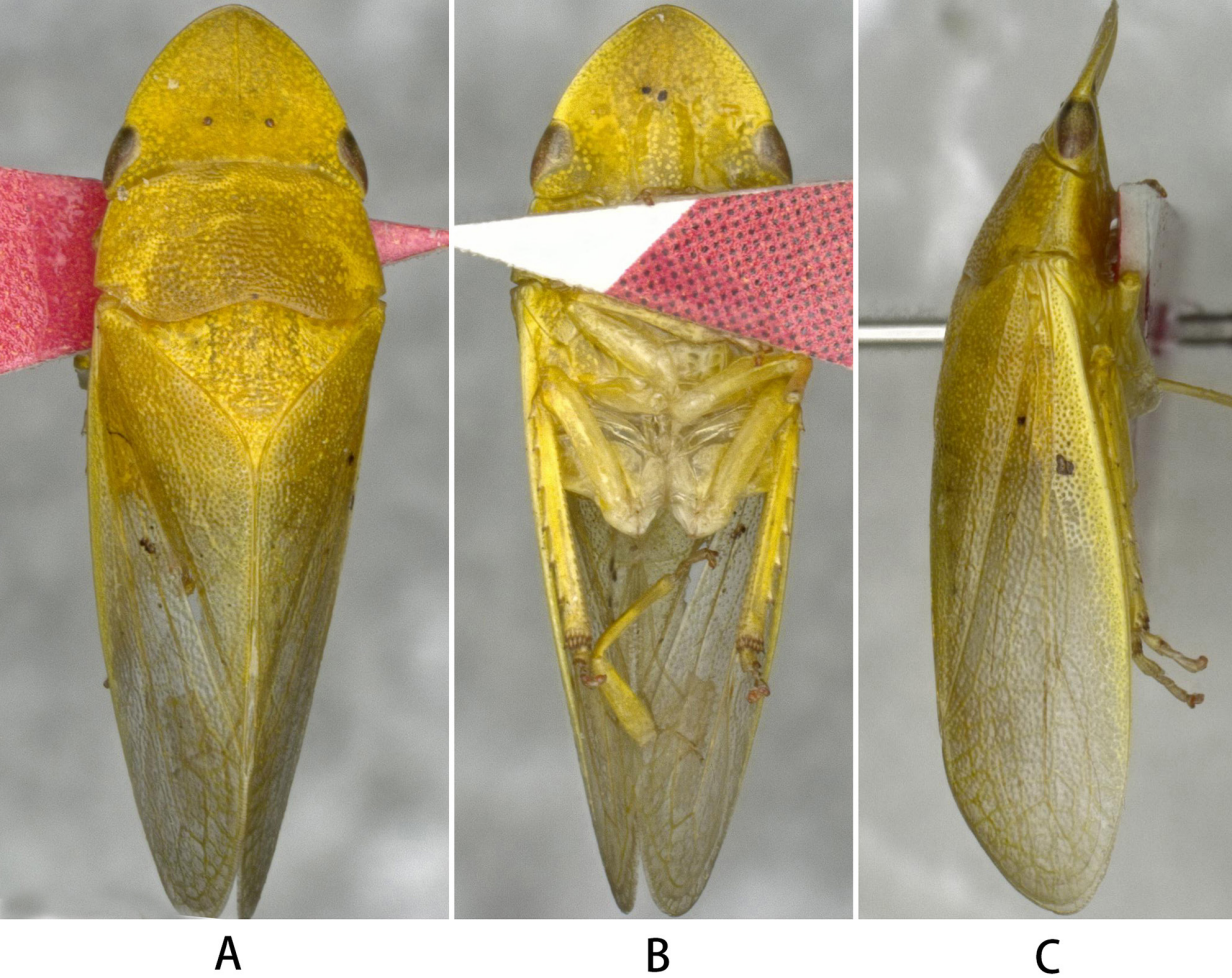
External morphology of *Mencius quinquespinosus* (Wang, Yang, Li & Li), comb. nov. **A**. Dorsal habitus; **B**. Ventral habitus; **C**. Lateral habitus.

**Figure 2.**
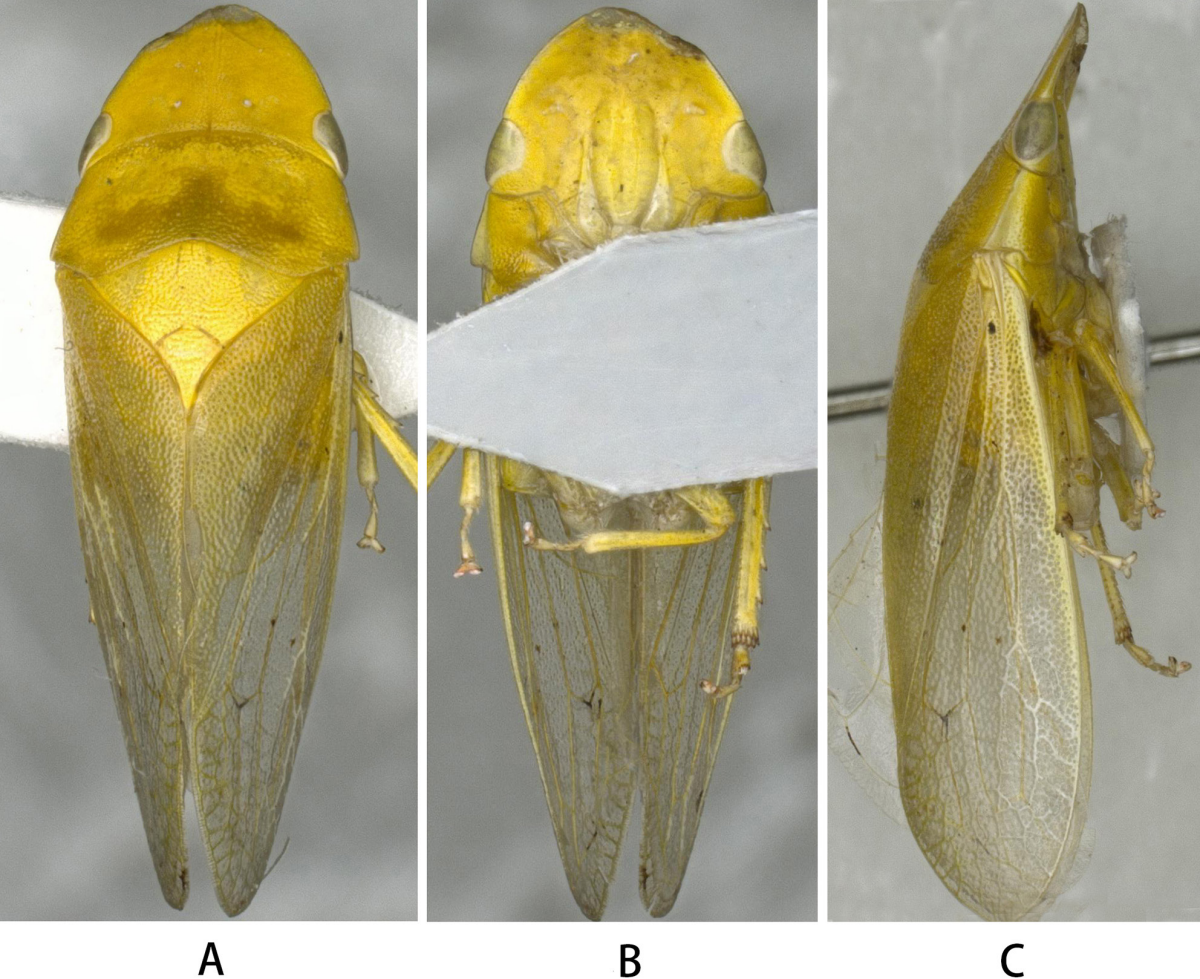
External morphology of *Mencius simplex* Li, Jiang & Dai, sp. nov. **A**. Dorsal habitus; **B**. Ventral habitus; **C**. Lateral habitus.

**Figure 3.**
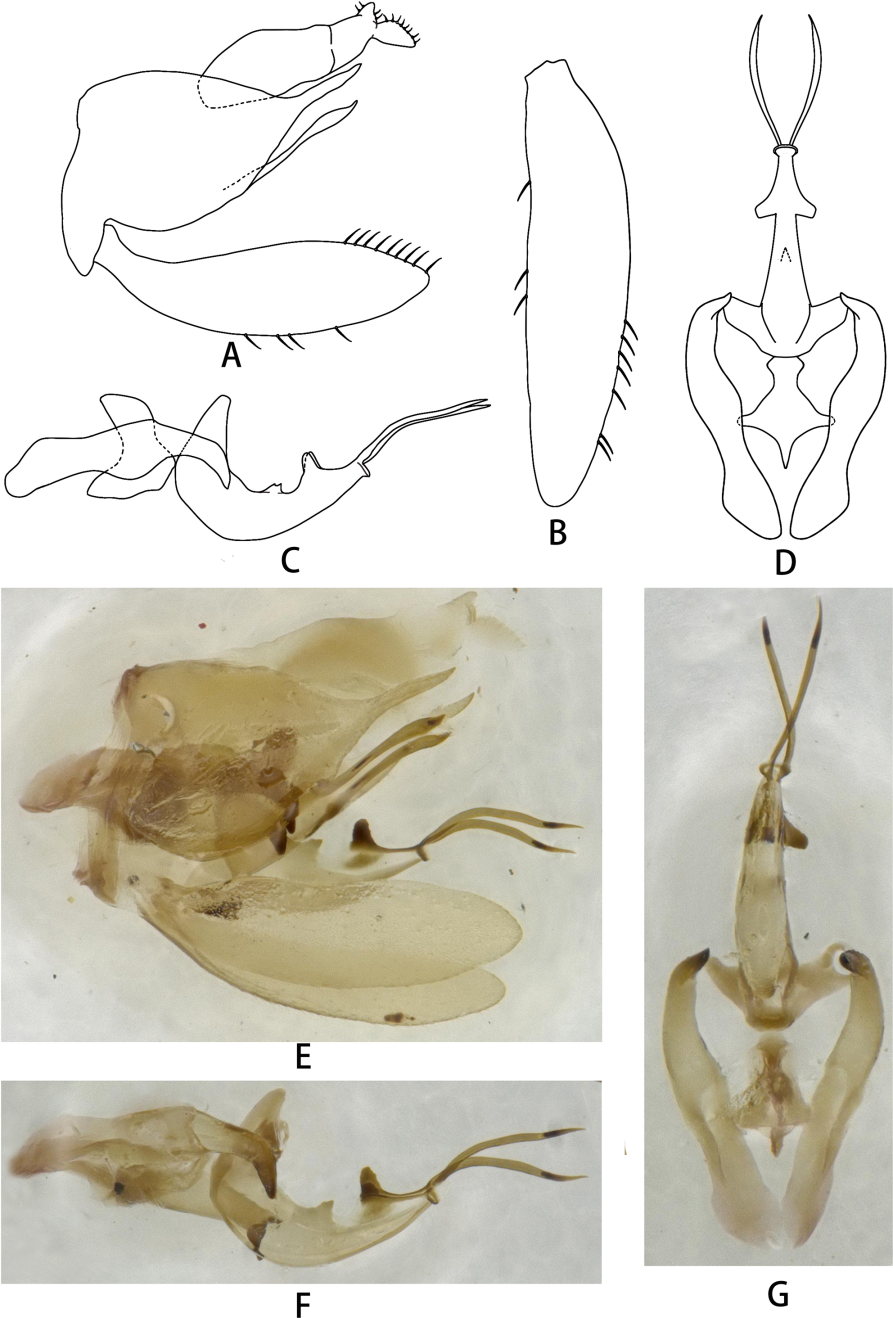
Male genitalia of *Mencius quinquespinosus* (Wang, Yang, Li & Li), comb. nov. **A, E**. Genital capsule, lateral view; **B**. Subgenital plate; **C, F**. Aedeagus, connective and style, lateral view; **D, G**. Aedeagus, connective and style, ventral view.

**Figure 4.**
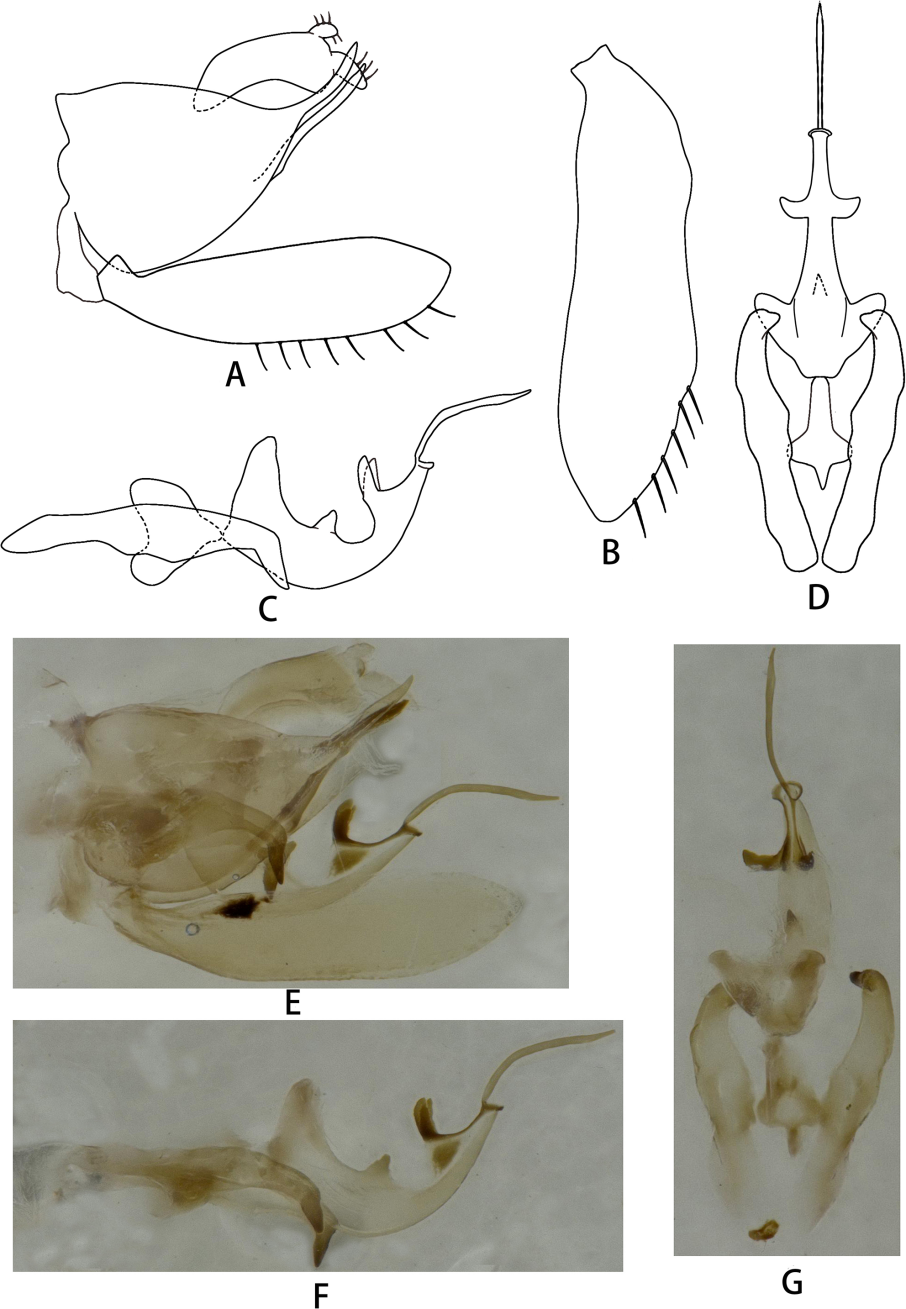
Male genitalia of *Mencius simplex* sp. nov. **A, E**. Genital capsule, lateral view; **B**. Subgenital plate; **C, F**. Aedeagus, connective and style, lateral view; **D, G**. Aedeagus, connective and style, ventral view.

**Figure 5.**
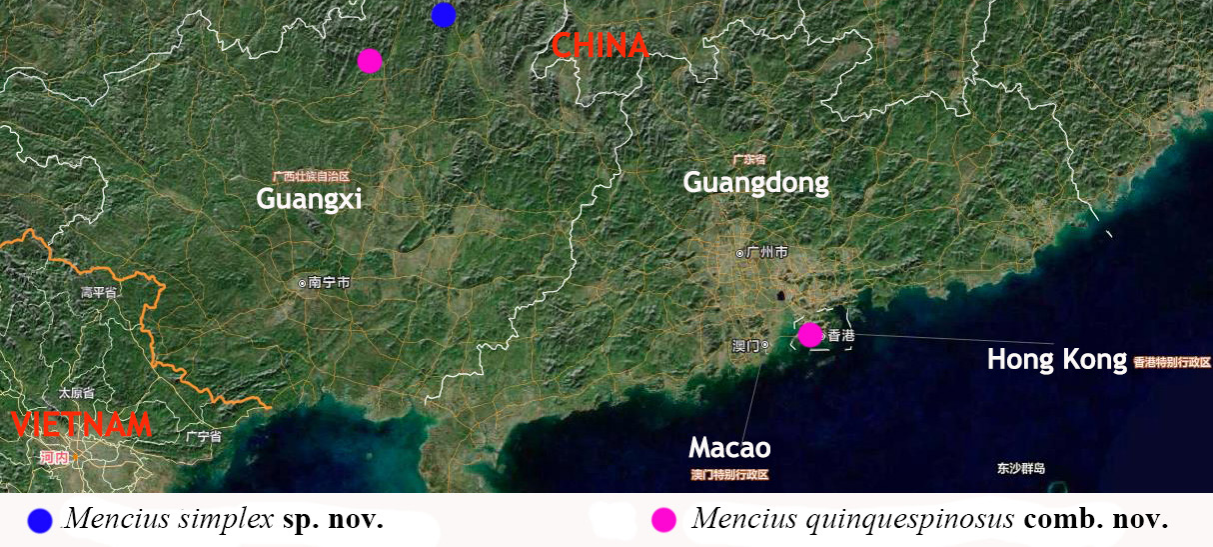
Distribution of *Mencius* gen. nov. in China.

##### Description.

Body large, length 10.0–15.0 mm (including tegmen); overall color yellowish green. Head (Figs 1A, 2A) clypeated, foliaceous produced anteriorly; vertex somewhat flattened; face beneath eyes strongly and abruptly narrowed, then gradually tapered, margins well defined; front small, narrow, flattish; eyes small; ocelli situated toward base of vertex, distance between ocelli less than distance from ocellus to eye. Pronotum (Figs 1A, 2A) transversely hexagonal, slightly wider than head, anteriorly narrowed; median length shorter than vertex; lateral margins acute, anterior lateral margins much longer than posterior lateral margins; anterior margin slightly rounded, posterior margin slightly concave. Scutellum (Figs 1A, 2A) triangular, subequilateral, nearly equal in length to pronotum, with distinct transverse depression. Anterior portion of body, in lateral view, appearing flexed ventrad from point posterior to scutellum. Forewings (Figs 1A, 1C, 2A, 2C) subcoriaceous, pellucid, densely punctate, tectiform; clavus broad before middle; corium obliquely rounded at apex; veins somewhat irregularly anastomosed toward apex. Hind legs with posterior tibiae remotely dentate.

***Male genitalia***. Pygofer (Figs 3A, 3E, 4A, 4E) with long ventrocaudal process, apex gradually tapered. Subgenital plates (Figs 3A, 3B, 3E, 4A, 4B, 4E) fused basally, elongate, inner margin with short spine-like setae. Aedeagus (Figs 3C, 3D, 3F, 3G, 4C, 4D, 4F, 4G) with shaft elongate, tubular, curved dorsally, with dorsal triangular protrusion near center; gonopore apical, orifice with 1 or 2 slender processes; basal apodeme distinct. Style (Figs 3C, 3D, 3F, 3G, 4C, 4D, 4F, 4G) with anterior portion approximately equal in length to caudal portion; caudal portion robust, apex strongly recurved. Connective (Figs 3C, 3D, 3F, 3G, 4C, 4D, 4F, 4G) T-shaped.

##### Diagnosis.

The new genus resembles *Petalocephala* Stål and *Platycephala* Kuoh but can be distinguished by the following combination of characters: male pygofer with long ventrocaudal process, apex gradually tapered; aedeagus with dorsal triangular protrusion near center, gonopore orifice with 1 or 2 slender processes.

##### Distribution.

China: Guangxi, Hong Kong (Fig. 5).

##### Etymology.

The generic name honors Mencius, the famous Chinese philosopher and representative of Confucian culture. The name is masculine in gender.

##### Remarks.

The new genus is placed in the tribe Ledrini based on the combination of the following characters: head clypeated, foliaceously produced anteriorly; forewings subcoriaceous, densely punctate; and posterior tibiae remotely dentate.

#### Mencius quinquespinosus

Taxon classification

Animalia

HemipteraCicadellidae

(Wang, Yang, Li & Li) comb. nov.

D311A660-0811-58FA-B365-8AAA9D0549FD

Figs 1, 3, 5

Petalocephala quinquespinosa Wang, Yang, Li & Li, 2024: 20 (figs 5S1–S3, 7S1–S3).

##### Description.

Head (Fig. 1A) yellowish green; eyes dark grey; ocelli grey. Thorax sordid yellowish green. Forewings (Fig. 1A, C) yellowish hyaline, semitransparent.

***Head***. Crown flat, more or less horizontal, surface punctate with median ridge, approximately 2.5 times as long as wide between eyes (Fig. 1A). Ocelli not prominent, closer to each other than to adjacent eye. Pronotum shallowly foveate on either side of median line in anterior half, posterior half slightly gibbous; anterior margin slightly convex, posterior margin medially concave, lateral margin somewhat straight; medially approximately same length as crown (Fig. 1A). Mesonotum equal in length to pronotum (Fig. 1A). Forewing with claval region densely punctate, apical margin obliquely truncate (Fig. 1A, C).

***Male genitalia***. Pygofer side triangular in lateral view, apex gradually tapered, with slender process extending posteriorly from ventral margin internally (Fig. 3A, E). Style broad in middle region, caudal portion approximately as long as anterior portion, tapering anteriorly and posteriorly; caudal portion robust, posterior portion curved ventrally with beak-shaped apex (Fig. 3C, D, F, G). Connective T-shaped with high dorsomedial keel (Fig. 3C, D, F, G). Aedeagus with shaft elongate, tubular, curved dorsally, with dorsal protrusion near center and pair of dorsal protrusions near first one-third; gonopore apical, orifice with 2 slender processes; basal apodeme distinct (Fig. 3C, D, F, G).

##### Measurements.

Length (including tegmen): ♂, 10.15–10.8 mm (*N* = 2).

##### Material examined.

China • ♂, Guangxi, Liuzhou, Rongshui, 14.vi.2023, coll. Liu Yongcheng (QFNU).

##### Host plant.

Unknown.

##### Distribution.

China: Guangxi (Fig. 5).

##### Etymology.

The specific epithet *quinquespinosa* is an adjective, and we have adjusted its gender to agree with the masculine generic name: *Mencius quinquespinosus*.

#### Mencius simplex

Taxon classification

Animalia

HemipteraCicadellidae

Li, Jiang & Dai sp. nov.

A05C783C-8CEB-5ABE-AF3F-5260D47957C3

https://zoobank.org/2922D682-6DAA-453F-B795-7E0FAFB30688

Figs 2, 4, 5

##### Description.

Head (Fig. 2A) yellowish green; eyes grey; ocelli grayish white. Thorax sordid yellowish green. Forewings (Fig. 2A, C) yellowish hyaline, semitransparent.

***Head***. Crown flat, more or less horizontal, surface punctate with median ridge, approximately 2.2 times as long as wide between eyes (Fig. 2A). Ocelli not prominent, positioned nearly equidistant from each other and from adjacent eye. Pronotum shallowly foveate on either side of median line in anterior half; anterior margin slightly convex, posterior margin medially concave, lateral margin somewhat straight; medially slightly shorter than crown (Fig. 2A). Mesonotum slightly longer than pronotum (Fig. 2A). Forewing with claval region densely punctate, apical margin obliquely truncate (Fig. 2A, C).

***Male genitalia***. Pygofer side triangular in lateral view, apex gradually tapered, with slender process extending posteriorly from ventral margin internally (Fig. 4A, E). Style broad in middle region, caudal portion shorter than anterior portion, tapering anteriorly and posteriorly; caudal portion robust, anterior portion curved ventrally with beak-shaped apex (Fig. 4C, D, F, G). Connective T-shaped with high dorsomedial keel (Fig. 4C, D, F, G). Aedeagus with shaft elongate, tubular, curved dorsally, with dorsal protrusion near center and pair of dorsal protrusions near first one-third; gonopore apical, orifice with 1 slender process; basal apodeme distinct (Fig. 4C, D, F, G).

##### Measurements.

Length (including tegmen): ♂, 11.2 mm (*N* = 1).

##### Type material.

***Holotype***: • ♂, China: Guangxi, Guilin, Huaping, 2.vii.2022, coll. Jiang Sai (QFNU).

##### Host plant.

Unknown.

##### Distribution.

China: Guangxi (Fig. 5).

##### Etymology.

The species name is derived from the Latin words simplex (simple), referring to the single process of the aedeagal shaft.

## Supplementary Material

XML Treatment for Mencius

XML Treatment for Mencius quinquespinosus

XML Treatment for Mencius simplex

## Additional information

### Artificial Intelligence (AI) use

The authors accept full responsibility for the content of the manuscript, including the disclosure of any use of AI.

No AI tools were used in the preparation of this manuscript.

### Author ORCIDs

Y.-J. Li https://orcid.org/0000-0002-0470-203X

L.-N. Jiang https://orcid.org/0000-0003-3217-9191

### Data availability

All of the data that support the findings of this study are available in the main text.
